# Monotonicity, Concavity, and Convexity of Fractional Derivative of Functions

**DOI:** 10.1155/2013/605412

**Published:** 2013-10-31

**Authors:** Xian-Feng Zhou, Song Liu, Zhixin Zhang, Wei Jiang

**Affiliations:** School of Mathematical Sciences, Anhui University, Hefei 230039, China

## Abstract

The monotonicity of the solutions of a class of nonlinear fractional differential equations is studied first, and the existing results were extended. Then we discuss monotonicity, concavity, and convexity of fractional derivative of some functions and derive corresponding criteria. Several examples are provided to illustrate the applications of our results.

## 1. Introduction and Preliminaries

Fractional calculus is a generalization of the traditional integer order calculus. Recently, fractional differential equations have received increasing attention since behavior of many physical systems can be properly described as fractional differential systems. Most of the present works focused on the existence, uniqueness, and stability of solutions for fractional differential equations, controllability and observability for fractional differential systems, numerical methods for fractional dynamical systems, and so on see the monographs [1–4] and the papers [5–24]. However, there existed a flaw in paper [6], which has been stated in paper [21]. The main reason that the flaw arose is that one is unknown of monotonicity, concavity, and convexity of fractional derivative of a function.

It is well known that the monotonicity, the concavity, and the convexity of a function play an important role in studying the sensitivity analysis for variational inequalities, variational inclusions, and complementarity. Since fractional derivative of a function is usually not an elementary function, its properties are more complicated than those of integer order derivative of the function. The focal point of this paper is to investigate the monotonicity, the concavity, and the convexity of fractional derivative of some functions.

Now we recall some definitions and lemmas which will be used later. For more detail, see [1–4].

**Definition 1**

Given an interval [*a*, *b*] of ℝ, the fractional order integral of a function *f* ∈ *L* ^1^[*a*, *b*] of order *α* ∈ ℝ^+^ is defined by

(1) Iaαf(t)=1Γ(α)∫at(t−s)α−1f(s)ds,t∈[a,b],  α>0,

where Γ is the Gamma function.

**Definition 2**

Riemann-Liouville's derivative of order *α* with the lower limit *a* for a function *f* ∈ *L* ^1^[*a*, *b*] can be written as

(2) RLDaαf(t)=1Γ(n−α)dndtn∫at(t−s)n−α−1f(s)ds,t∈[a,b],  0<n−1≤α<n.

**Definition 3**

Suppose that a function *f* is defined on the interval [*a*, *b*] and *f* ^(*n*)^(*t*) ∈ *L* ^1^[*a*, *b*]. The Caputo's fractional derivative of order *α* with lower limit *a* for *f* is defined as

(3)   CDaαf(t)=1Γ(n−α)∫at(t−s)n−α−1f(n)(s)ds=Ian−αf(n)(t),   t∈[a,b],

where 0 < *n* − 1 < *α* ≤ *n*.

Particularly, when 0 < *α* ≤ 1, it holds that

(4)   CDaαf(t)=1Γ(1−α)∫at(t−s)−αf˙(s)ds=Ia1−αf˙(t),   t∈[a,b].

**Lemma 4**

There exists a link between Riemann-Liouville and Caputo's fractional derivative of order *α*. Namely,

(5)   CDaαf(t)=RLDaαf(t)−∑k=0n−1f(k)(a)Γ(k−α+1)(t−a)k−α,t>a,  n−1<Re(α)<n,

where *Re*(*α*) denotes the real parts of *α*.

Particularly, for 0 < *α* < 1, it holds that

(6)   CDaαf(t)=1Γ(1−α)∫at(t−s)−αf′(s)ds=DRLaαf(t)−f(a)Γ(1−α)(t−a)−α, t>a.

**Definition 5**

A function *f* : [*a*, *b*] → ℝ with [*a*, *b*] ⊂ ℝ is said to be convex if whenever *t*_1_ ∈ [*a*, *b*], *t*_2_ ∈ [*a*, *b*], and *θ* ∈ [0,1], the inequality

(7) $$\begin{array}{c} f\left(\theta t_{1}+\left(1-\theta \right)t_{2}\right)\leq \theta f\left(t_{1}\right)+\left(1-\theta \right)f\left(t_{2}\right) \end{array}$$

holds.

The rest of this paper is organized as follows.  Section 2 is devoted to monotonicity of solutions of fractional differential equations. In Section 3, we present the monotonicity, the concavity, and the convexity of functions ^RL^ *D*_*t*_0__^*α*^ *f*(*t*) and ^*C*^ *D*_*t*_0__^*α*^ *f*(*t*). Summarizing this paper forms the content of Section 4.

## 2. Monotonicity of Solutions of Nonlinear Fractional Differential Equations

In this section, we mainly investigate the monotonicity of the solution of nonlinear fractional differential equation with Caputo's derivative

(8) $$\begin{array}{c} {\;}^{C}D_{t_{0}}^{\alpha }u\left(t\right)=g\left(t,u\left(t\right)\right),\;\;t\geq t_{0}, \end{array}$$

which was discussed in [6, 21], where 0 < *α* < 1. The paper [21] gave two examples to show that Lemma  1.7.3 in [6] is invalid. Lemma  1.7.3 in [6] is as follows.

Lemma  1.7.3 in [6] consider (8), where 0 < *α* < 1 and *g*(*t*, *u*) ≥ 0. Then, if the solutions exist, they are nondecreasing in *t*.

In [21], the authors gave an improvement of Lemma 1.7.3, which is as follows.

Lemma  2.4 in [21] consider (8). Suppose that 0 < *α* < 1, *g*(*t*, *u*) ≥ 0, and *t*_0_ ∈ ℝ. If the solutions exists and *u*(*t*_0_) ≥ 0, then they are nonnegative. Furthermore, If *g*(*t*, *u*) = *λu* for *λ* > 0, then the solutions are nondecreasing in *t*.

Now we will give a more general result for (8), which is an improvement of Lemma  2.4 in [21].

**Theorem 6**

Assume that 0 < *α* < 1. Assume that the solutions of (8) exist.

1. If *g*(*t*, *u*) ≥ 0 on [*t*_0_, *t*_1_] and *u*(*t*_0_) ≥ 0, then the solutions *u*(*t*) of (8) are nonnegative on [*t*_0_, *t*_1_].
2. If *g*(*t*_0_, *u*(*t*_0_)) ≥ 0 and (*d*/*dt*)*g*(*t*, *u*(*t*)) ≥ 0 on [*t*_0_, *t*_1_], then the solution *u*(*t*) of (8) is nondecreasing on [*t*_0_, *t*_1_].
3. If *g*(*t*_0_, *u*(*t*_0_)) ≤ 0 and (*d*/*dt*)*g*(*t*, *u*(*t*)) ≤ 0 on [*t*_0_, *t*_1_], then the solution *u*(*t*) of (8) is not increasing on [*t*_0_, *t*_1_].

**Proof**

The conclusion of (1) is obvious. In fact, (8) is equivalent to

(9) $$\begin{array}{c} u\left(t\right)=u\left(t_{0}\right)+\frac{1}{\Gamma \left(\alpha \right)}\overset{t}{\underset{t_{0}}{\int }}{\left(t-s\right)}^{\alpha -1}g\left(s,u\left(s\right)\right)ds. \end{array}$$

Since *g*(*t*, *u*(*t*)) ≥ 0, it holds that (1/Γ(*α*))∫_*t*_0__^*t*^(*t* − *s*)^*α*−1^ *g*(*s*, *u*(*s*))*ds* ≥ 0. Noting *u*(*t*_0_) ≥ 0, we have *u*(*t*) ≥ 0.

Now we prove the validity of (2) and (3). First, by the definition of the Caputo's derivative, it holds from (8) that

(10) $$\begin{array}{c} I_{t_{0}}^{1-\alpha }\left(\dot{u}\left(t\right)\right)=g\left(t,u\left(t\right)\right). \end{array}$$

Then it follows that

(11) $$\begin{array}{c} I_{t_{0}}^{\alpha }\left(I_{t_{0}}^{1-\alpha }\dot{u}\left(t\right)\right)=I_{t_{0}}^{\alpha }\left(g\left(t,u\left(t\right)\right)\right). \end{array}$$

That is,

(12) $$\begin{array}{c} I_{t_{0}}^{1}\left(\dot{u}\left(t\right)\right)=I_{t_{0}}^{\alpha }\left(g\left(t,u\left(t\right)\right)\right). \end{array}$$

Then we can get that

(13) u˙(t)=ddt(It0αg(t,u(t)))= RLDt01−α(g(t,u(t)))= CDt01−α(g(t,u(t)))+g(t0,u(t0))Γ(1−α)(t−t0)−α=It0α(ddtg(t,u(t)))+g(t0,u(t0))Γ(1−α)(t−t0)−α,t∈[t0,t1].

Since (*d*/*dt*)*g*(*t*, *u*(*t*)) ≥ 0 on [*t*_0_, *t*_1_] and *g*(*t*_0_, *u*(*t*_0_)) ≥ 0, thus $\dot{u}(t)\geq 0$ on [*t*_0_, *t*_1_]. Hence *u*(*t*) is nondecreasing on [*t*_0_, *t*_1_], and (2) holds.

Similar to the proof of (2), we can prove that (3) holds. This completes the proof.

**Remark 7**

Lemma  2.4 in [21] is a particular case of Theorem 6 of this paper. In fact in Lemma  2.4 in [21], if *g*(*t*, *u*) = *λu*, then (8) is ^*C*^ *D*_*t*_0__^*α*^ *u*(*t*) = *λu*. The solution *u*(*t*) of ^*C*^ *D*_*t*_0__^*α*^ *u*(*t*) = *λu* is

(14) $$\begin{array}{c} u\left(t\right)=u\left(t_{0}\right)E_{\alpha ,1}\left(\lambda {\left(t-t_{0}\right)}^{\alpha }\right),\;\;t\geq t_{0}. \end{array}$$

If *u*(*t*_0_) ≥ 0, then

(15) g(t,u)=λu=λu(t0)Eα,1(λ(t−t0)α)≥0,  t≥t0,ddtg(t,u)=λ2u(t0)(t−t0)α−1Eα,α(λ(t−t0)α)≥0,

which satisfies the conditions of (2) in Theorem 6.

**Example 8**

Assume that 0 < *α* < 1. Consider the fractional differential equation

(16) $$\begin{array}{c} {}^{C}D_{0}^{\alpha }u\left(t\right)=t+\sin t,\;\;t\geq 0. \end{array}$$

For *t* > 0, we have *t* + sin*t* ≥ 0 and (*t* + sin*t*)′ = 1 − cos⁡*t* ≥ 0. By Theorem 6, we see that *u*(*t*) is nondecreasing in *t* for *t* ≥ 0.

**Example 9**

Assume that 0 < *α* < 1. Consider the fractional differential equation

(17) $$\begin{array}{c} {}^{C}D_{t_{0}}^{\alpha }f\left(t\right)=-2,\;\;t\geq t_{0}. \end{array}$$

Denote *g*(*t*, *f*(*t*)) = −2, then *g*(*t*_0_, *f*(*t*_0_)) < 0 and $\dot{g}(t,f(t))=0$ for *t* ≥ *t*_0_. By Theorem 6, it follows that *f*(*t*) is not increasing. In fact, by computation we get $\dot{f}(t)=(-2\alpha /\Gamma (1+\alpha ))t^{\alpha -1}<0$ on [*t*_0_, *∞*), thus *f*(*t*) is decreasing.

The following fractional comparison principle is an improvement of Lemma  6.1 in [20] and Theorem  2.6 in [21]. The method we used here is different from the one used to prove Lemma  6.1 in [20] and the one used to prove Theorem  2.6 in [21].

**Theorem 10**

Suppose that 0 < *α* < 1 and ^*C*^ *D*_*t*_0__^*α*^ *f*(*t*) ≥ ^*C*^ *D*_*t*_0__^*α*^ *g*(*t*) on interval [*t*_0_, *t*_1_]. Suppose further that *f*(*t*_0_) ≥ *g*(*t*_0_), then *f*(*t*) ≥ *g*(*t*) on [*t*_0_, *t*_1_].

**Proof**

Set ^*C*^ *D*_*t*_0__^*α*^ *f*(*t*) − ^*C*^ *D*_*t*_0__^*α*^ *g*(*t*) = *m*(*t*), *t* ∈ [*t*_0_, *t*_1_]. Then

(18) $$\begin{array}{c} {}^{C}D_{t_{0}}^{\alpha }\left(f\left(t\right)-g\left(t\right)\right)=m\left(t\right)\geq 0,\;\;t\in \left[t_{0},t_{1}\right]. \end{array}$$

Taking *I*_*t*_0__^*α*^ on both sides of (18) yields

(19) $$\begin{array}{c} I_{t_{0}}^{\alpha }\left({}^{C}D_{t_{0}}^{\alpha }\left(f\left(t\right)-g\left(t\right)\right)\right)=I_{t_{0}}^{\alpha }\left(m\left(t\right)\right). \end{array}$$

That is,

(20) $$\begin{array}{c} f\left(t\right)-g\left(t\right)=f\left(t_{0}\right)-g\left(t_{0}\right)+I_{t_{0}}^{\alpha }\left(m\left(t\right)\right). \end{array}$$

Since *m*(*t*) ≥ 0, thus *I*_*t*_0__^*α*^(*m*(*t*)) ≥ 0. Then we have

(21) $$\begin{array}{c} f\left(t\right)-g\left(t\right)\geq f\left(t_{0}\right)-g\left(t_{0}\right)\geq 0,\;\;t\in \left[t_{0},t_{1}\right]. \end{array}$$

Hence *f*(*t*) ≥ *g*(*t*) on [*t*_0_, *t*_1_], and the proof is completed.

**Remark 11**

The method used to prove Theorem  2.6 in [21] and to prove Lemma  6.1 in [20] is the Laplace transform, which demands *t* ∈ [0, *∞*). Theorem  2.6 in [21] and Lemma  6.1 in [20] are as follows, respectively.

Theorem  2.6 in [21] suppose that 0 < *α* < 1 and ^*C*^ *D*_0_^*α*^ *v*(*t*) ≥ ^*C*^ *D*_0_^*α*^ *w*(*t*) on ℝ_+_. If *v*(0) ≥ *w*(0), then *v*(*t*) ≥ *w*(*t*) on ℝ_+_.

Lemma  6.1 in [20] let ^*C*^ *D*_0_^*β*^ *x*(*t*) ≥ ^*C*^ *D*_0_^*β*^ *y*(*t*) and *x*(0) = *y*(0), where *β* ∈ (0,1). Then *x*(*t*) ≥ *y*(*t*).

## 3. Monotonicity, Concavity, and Convexity of the Functions ^RL^ *D*_*t*_0__^*α*^ *f*(*t*) and ^*C*^ *D*_*t*_0__^*α*^ *f*(*t*)

In this section, we first investigate the monotonicity of the functions ^RL^ *D*_*t*_0__^*α*^ *f*(*t*) and ^*C*^ *D*_*t*_0__^*α*^ *f*(*t*).

**Theorem 12**

Assume that 0 < *α* < 1. If there exists an interval [*t*_0_, *t*_1_] such that

1. *f*(*t*_0_) ≤ 0, $\dot{f}(t_{0})\geq 0$, and $\ddot{f}(t)\geq 0$ on [*t*_0_, *t*_1_], then ^*RL*^ *D*_*t*_0__^*α*^ *f*(*t*) is nondecreasing on [*t*_0_, *t*_1_];
2. *f*(*t*_0_) ≥ 0, $\dot{f}(t_{0})\leq 0$, and $\ddot{f}(t)\leq 0$ on [*t*_0_, *t*_1_], then ^*RL*^ *D*_*t*_0__^*α*^ *f*(*t*) is not increasing on [*t*_0_, *t*_1_];
3. *f*(*t*_0_) > 0, $\dot{f}(t_{0})>0$, and $\ddot{f}(t)\in C([t_{0},t_{1}],(0,+\infty ))$ (i.e., $\ddot{f}(t)$ is continuous on [*t*_0_, *t*_1_] and $\ddot{f}(t)>0$), then there exists a constant *β* ∈ [*t*_0_, *t*_1_] such that ^*RL*^ *D*_*t*_0__^*α*^ *f*(*t*) is not increasing on [*t*_0_, *β*] and is not decreasing on [*β*, *t*_1_];
4. *f*(*t*_0_) < 0, $\dot{f}(t_{0})<0$, and $\ddot{f}(t)\in C([t_{0},t_{1}],(-\infty ,0))$, then there exists a constant *η* ∈ [*t*_0_, *t*_1_] such that ^*RL*^ *D*_*t*_0__^*α*^ *f*(*t*) is nondecreasing on [*t*_0_, *η*] and ^*RL*^ *D*_*t*_0__^*α*^ *f*(*t*) is not increasing on [*η*, *t*_1_].

**Proof**

Using formula (6), we have

(22) RLDt0αf(t)=DCt0αf(t)+f(t0)Γ(1−α)(t−t0)−α=1Γ(1−α)∫t0t(t−s)−αf˙(s)ds +f(t0)Γ(1−α)(t−t0)−α.

Then we can get that

(23) ddt(RLDt0αf(t)) =ddt(CDt0αf(t))−αf(t0)Γ(1−α)(t−t0)−α−1 =ddt(It01−αf˙(t))−αf(t0)Γ(1−α)(t−t0)−α−1 =RLDt0α(f˙(t))−αf(t0)Γ(1−α)(t−t0)−α−1 =CDt0αf˙(t)+f˙(t0)Γ(1−α)(t−t0)−α  −αf(t0)Γ(1−α)(t−t0)−α−1 =1Γ(1−α)∫t0t(t−s)−αf¨(s)ds+f˙(t0)Γ(1−α)(t−t0)−α  −αf(t0)Γ(1−α)(t−t0)−α−1.

By assumptions in (1), it follows that (*d*/*dt*)(^RL^ *D*_*t*_0__^*α*^ *f*(*t*)) ≥ 0 on [*t*_0_, *t*_1_]. Thus ^RL^ *D*_*t*_0__^*α*^ *f*(*t*) is nondecreasing on [*t*_0_, *t*_1_]. By assumptions in (2), it follows that ^RL^ *D*_*t*_0__^*α*^ *f*(*t*) is not increasing in *t* on [*t*_0_, *t*_1_]. Consequently, the conclusions of (1) and (2) are true.

Let us prove (3). Noting formula (23),

(24) ddt(RLDt0αf(t)) =1Γ(1−α)∫t0t(t−s)−αf¨(s)ds+f˙(t0)Γ(1−α)(t−t0)−α  −αf(t0)Γ(1−α)(t−t0)−α−1.

Since *f*(*t*_0_) > 0 and $\dot{f}(t_{0})>0$, then

(25) $$\begin{array}{c} \frac{\dot{f}\left(t_{0}\right)}{\Gamma \left(1-\alpha \right)}{\left(t-t_{0}\right)}^{-\alpha }-\frac{\alpha f\left(t_{0}\right)}{\Gamma \left(1-\alpha \right)}{\left(t-t_{0}\right)}^{-\alpha -1}\to -\infty \end{array}$$

as *t* → *t*_0_. By the fact that $\ddot{f}(t)\in C([t_{0},t_{1}],(0,+\infty ))$, we have

(26) $$\begin{array}{c} \frac{1}{\Gamma \left(1-\alpha \right)}\overset{t}{\underset{t_{0}}{\int }}{\left(t-s\right)}^{-\alpha }\ddot{f}\left(s\right)ds\to 0,\;\;t\to t_{0}. \end{array}$$

Thus there exists a constant *δ*_1_ > 0 such that (*d*/*dt*)(^RL^ *D*_*t*_0__^*α*^ *f*(*t*)) ≤ 0 on [*t*_0_, *t*_0_ + *δ*_1_]. On the other hand, when $t\geq t_{0}+\alpha f\left(t_{0}\right)/\dot{f}\left(t_{0}\right)$,

(27) $$\begin{array}{c} \frac{\dot{f}\left(t_{0}\right)}{\Gamma \left(1-\alpha \right)}{\left(t-t_{0}\right)}^{-\alpha }-\frac{\alpha f\left(t_{0}\right)}{\Gamma \left(1-\alpha \right)}{\left(t-t_{0}\right)}^{-\alpha -1}\geq 0. \end{array}$$

Thus there exists a constant *β* ∈ [*t*_0_, *t*_1_] such that (*d*/*dt*)(^RL^ *D*_*t*_0__^*α*^ *f*(*t*)) ≤ 0 on [*t*_0_, *β*] and (*d*/*dt*)(^RL^ *D*_*t*_0__^*α*^ *f*(*t*)) ≥ 0 on [*β*, *t*_1_]. Therefore, the conclusion of (3) is valid.

The proof of (4) is similar to that of (3). This completes the proof.

Now we are to investigate the monotonicity of the function ^*C*^ *D*_*t*_0__^*α*^ *f*(*t*).

**Theorem 13**

Assume that 0 < *α* < 1. If there exists an interval [*t*_0_, *t*_1_] such that $\ddot{f}(t)\geq 0$ on [*t*_0_, *t*_1_] and $\dot{f}(t_{0})\geq 0$, then ^*C*^ *D*_*t*_0__^*α*^ *f*(*t*) is nondecreasing on [*t*_0_, *t*_1_]. If $\ddot{f}(t)\leq 0$ on [*t*_0_, *t*_1_] and $\dot{f}(t_{0})\leq 0$, then ^*C*^ *D*_*t*_0__^*α*^ *f*(*t*) is not increasing on [*t*_0_, *t*_1_].

**Proof**

Set $\dot{f}(t)=g(t)$. Note that

(28) ddt(CDt0αf(t))=ddt(It01−αf˙(t))=ddt(It01−αg(t))=RLDt0αg(t)=CDt0αg(t)+g(t0)Γ(1−α)(t−t0)−α=1Γ(1−α)∫t0t(t−u)−αg˙(u)du +g(t0)Γ(1−α)(t−t0)−α.

If $\dot{g}(t)=\ddot{f}(t)\geq 0$ on [*t*_0_, *t*_1_] and $g(t_{0})=\dot{f}(t_{0})\geq 0$, then (*d*/*dt*)(^*C*^ *D*_*t*_0__^*α*^ *f*(*t*)) ≥ 0 in *t* on [*t*_0_, *t*_1_]. Hence, ^*C*^ *D*_*t*_0__^*α*^ *f*(*t*) is nondecreasing on interval [*t*_0_, *t*_1_]. If $\dot{g}(t)=\ddot{f}(t)\leq 0$ and $g(t_{0})=\dot{f}(t_{0})\leq 0$, then (*d*/*dt*)(^*C*^ *D*_*t*_0__^*α*^ *f*(*t*)) ≤ 0. Hence, ^*C*^ *D*_*t*_0__^*α*^ *f*(*t*) is not increasing on [*t*_0_, *t*_1_]. The proof is completed.

The following examples illustrate applications of Theorems 12 and 13.

**Example 14**

Assume that 0 < *α* < 1. Consider ^RL^ *D*_*t*_0__^*α*^ *f*(*t*), where *f*(*t*) = *e* ^*t*^, for all *t*_0_ ∈ ℝ. Since $f(t_{0})=\dot{f}(t_{0})>0$ and $\ddot{f}(t)\in C((-\infty ,+\infty ),(0,+\infty ))$, by Theorem 12, there exists a constant *β* > *t*_0_ such that ^RL^ *D*_*t*_0__^*α*^(*e* ^*t*^) is decreasing on [*t*_0_, *β*] and is increasing on [*β*, +*∞*).

**Example 15**

Assume that 0 < *α* < 1. Consider ^*C*^ *D*_0.5*π*_^*α*^sin*t* for *t* ∈ [*π*/2, *π*]. Since (sin*t*)′′ ≤ 0 for *t* ∈ [*π*/2, *π*] and (sin*t*)′|_*t*=*π*/2_ = 0, by Theorem 13, ^*C*^ *D*_0.5*π*_^*α*^sin*t* is decreasing on [*π*/2, *π*]. By similar argument, ^*C*^ *D*_1.5*π*_^*α*^sin*t* is increasing on *t* ∈ [3*π*/2,2*π*]. Since ^*C*^ *D*_*t*_0__^*α*^sin*t* = (1/Γ(1 − *α*))∫_*t*_0__^*t*^(*t* − *τ*)^−*α*^cos⁡*τd*, thus (1/Γ(1 − *α*))∫_0.5*π*_^*t*^(*t* − *τ*)^−*α*^cos⁡*τd* *τ* is decreasing on [*π*/2, *π*] and (1/Γ(1 − *α*))∫_1.5*π*_^*t*^(*t* − *τ*)^−*α*^cos⁡*τd* *τ* is increasing on *t* ∈ [3*π*/2,2*π*].

**Example 16**

Assume that 0 < *α* < 1. Consider ^*C*^ *D*_*t*_0__^*α*^ *f*(*t*); here *t*_0_ = 1 and *f*(*t*) = *t* − *t* ^2^. Obviously, $\dot{f}(t)=1-2t$ and $\ddot{f}(t)=-2$. For *t* ∈ [1, *∞*], $\dot{f}(1)=1-2<0$ and $\ddot{f}(t)=-2<0$. By Theorem 13, ^*C*^ *D*_*t*_0__^*α*^ *f*(*t*) is not increasing on [1, *∞*].

Next we are to investigate the concavity and the convexity of ^RL^ *D*_*t*_0__^*α*^ *f*(*t*) and ^*C*^ *D*_*t*_0__^*α*^ *f*(*t*). By formula (23), we have

(29) d2dt2(RLDt0αf(t)) =ddt(1Γ(1−α)∫t0t(t−s)−αf¨(s)ds+1Γ(1−α)(t−t0)−αf˙(t0)−α1Γ(1−α)(t−t0)−α−1f(t0)) =1Γ(1−α)∫t0t(t−s)−αf′′′(s)ds  +1Γ(1−α)(t−t0)−αf¨(t0)  −α1Γ(1−α)(t−t0)−α−1f˙(t0)  +α(α+1)1Γ(1−α)(t−t0)−α−2f(t0).

Thus we can obtain the following theorem.

**Theorem 17**

Assume that 0 < *α* < 1. If there exists an interval [*t*_0_, *t*_1_] such that *f*′′′(*t*) ≥ 0 on [*t*_0_, *t*_1_], $\ddot{f}(t_{0})\geq 0$, $\dot{f}(t_{0})\leq 0$ and *f*(*t*_0_) > 0, then ^*RL*^ *D*_*t*_0__^*α*^ *f*(*t*) is concave on [*t*_0_, *t*_1_]. If *f*′′′(*t*) ≤ 0 on [*t*_0_, *t*_1_], $\ddot{f}(t_{0})\leq 0$ and $\dot{f}(t_{0})\geq 0$ and *f*(*t*_0_) ≤ 0, then ^*RL*^ *D*_*t*_0__^*α*^ *f*(*t*) is convex on [*t*_0_, *t*_1_].

The next theorem is on the convexity and the concavity of ^*C*^ *D*_*t*_0__^*α*^ *f*(*t*).

**Theorem 18**

Assume that 0 < *α* < 1. If there exists an interval [*t*_0_, *t*_1_] such that *f*′′′(*t*) ≥ 0 on [*t*_0_, *t*_1_], $\ddot{f}(t_{0})\leq 0$ and $\dot{f}(t_{0})\geq 0$, then ^*C*^ *D*_*t*_0__^*α*^ *f*(*t*) is concave on [*t*_0_, *t*_1_]. If *f*′′′(*t*) ≤ 0 on [*t*_0_, *t*_1_], $\ddot{f}(t_{0})\geq 0$ and $\dot{f}(t_{0})\geq 0$, then ^*C*^ *D*_*t*_0__^*α*^ *f*(*t*) is convex on [*t*_0_, *t*_1_].

**Proof**

By formula (28), we have

(30) d2dt2(CDt0αf(t))=ddt(CDt0αf˙(t)+f˙(t0)Γ(1−α)(t−t0)−α)  =ddt(It01−α(f¨(t)))−αf˙(t0)Γ(1−α)(t−t0)−α−1=RLDt0α(f¨(t))−αf˙(t0)Γ(1−α)(t−t0)−α−1=CDt0α(f¨(t))+f¨(t0)Γ(1−α)(t−t0)−α −αf˙(t0)Γ(1−α)(t−t0)−α−1=1Γ(1−α)∫t0t(t−s)−αf′′′(s)ds+f¨(t0)Γ(1−α)(t−t0)−α −αf˙(t0)Γ(1−α)(t−t0)−α−1.

If *f*′′′(*t*) ≥ 0 on [*t*_0_, *t*_1_], $\ddot{f}(t_{0})\geq 0$, and $\dot{f}(t_{0})\leq 0$, then (*d* ^2^/*dt* ^2^)(^*C*^ *D*_*t*_0__^*α*^ *f*(*t*)) ≥ 0 on [*t*_0_, *t*_1_]. Hence, ^*C*^ *D*_*t*_0__^*α*^ *f*(*t*) is concave in *t* on [*t*_0_, *t*_1_]. If *f*′′′(*t*) ≤ 0, on [*t*_0_, *t*_1_], $\ddot{f}(t_{0})\leq 0$, and $\dot{f}(t_{0})\geq 0$, then (*d* ^2^/*dt* ^2^)(^*C*^ *D*_*t*_0__^*α*^ *f*(*t*)) ≤ 0 on [*t*_0_, *t*_1_]. Therefore ^*C*^ *D*_*t*_0__^*α*^ *f*(*t*) is convex in *t* on [*t*_0_, *t*_1_].

**Example 19**

Assume that 0 < *α* < 1. Consider the fractional differential equation ^*C*^ *D*_*t*_0__^*α*^ *f*(*t*); here *t*_0_ < 0 and *f*(*t*) = *t* − *t* ^2^. Obviously, $\dot{f}(t)=1-2t$, $\ddot{f}(1)=-2$ and *f*′′′(*t*) = 0. For all *t*_0_ < 0, it holds that $\dot{f}(t_{0})>0$, $\ddot{f}(t_{0})<0$, and *f*′′′(*t*) = 0 on [*t*_0_, 0.5]. Then by Theorem 18, ^*C*^ *D*_*t*_0__^*α*^(*t* − *t* ^2^) is convex on [*t*_0_, 0.5].

**Example 20**

Consider the concavity and convexity of the function ^RL^ *D*_*t*_0__^*α*^ *f*(*t*), where *f*(*t*) = *e* ^*t*^, *t*_0_ ∈ ℝ. Obviously, Theorems 17 and 18 are useless to the function ^RL^ *D*_*t*_0__^*α*^ *e* ^*t*^. Now we employ the method which is used in the proof of Theorem 17 to investigate it. By formula (29), we have

(31) d2dt2(RLDt0αet) =1Γ(1−α)∫t0t(t−s)−αesds+1Γ(1−α)(t−t0)−αet0  −α1Γ(1−α)(t−t0)−α−1et0  +α(α+1)1Γ(1−α)(t−t0)−α−2et0.

The three terms in the right side of (31) can be reduced to

(32) 1Γ(1−α)(t−t0)−αet0−α1Γ(1−α)(t−t0)−α−1et0  +α(α+1)1Γ(1−α)(t−t0)−α−2et0 =1Γ(1−α)(t−t0)−α−2  ×et0[(t−t0)2−α(t−t0)+α(α−1)].

It is not difficult to get

(33) $$\begin{array}{c} {\left(t-t_{0}\right)}^{2}-\alpha \left(t-t_{0}\right)+\alpha \left(\alpha -1\right)\geq 0 \end{array}$$

for *t* ∈ [*t*_0_ +  (*α* + (4*α*−3*α* ^2^)^0.5^)/2, +*∞*) and

(34) $$\begin{array}{c} {\left(t-t_{0}\right)}^{2}-\alpha \left(t-t_{0}\right)+\alpha \left(\alpha -1\right)\leq 0 \end{array}$$

for *t* ∈ [*t*_0_, *t*_0_ + (*α* + (4*α*−3*α* ^2^)^0.5^)/2]. Thus (*d* ^2^/*dt* ^2^)(^RL^ *D*_*t*_0__^*α*^ *e* ^*t*^) > 0 on [*t*_0_ + (*α* + (4*α*−3*α* ^2^)^0.5^)/2, +*∞*). Consequently, ^RL^ *D*_*t*_0__^*α*^ *e* ^*t*^ is concave on [*t*_0_ + (*α* + (4*α*−3*α* ^2^)^0.5^)/2, *∞*). Since (1/Γ(1 − *α*))∫_*t*_0__^*t*^(*t* − *s*)^−*α*^ *e* ^*s*^ *ds* → 0 as *t* → *t*_0_, and (1/Γ(1 − *α*))∫_*t*_0__^*t*^(*t* − *s*)^−*α*^ *e* ^*s*^ *ds* is increasing on [*t*_0_, +*∞*), thus there exists a constant *β* ∈ (*t*_0_, *t*_0_ + (*α* + (4*α*−3*α* ^2^)^0.5^)/2) such that (*d* ^2^/*dt* ^2^)(^RL^ *D*_*t*_0__^*α*^ *e* ^*t*^) < 0 on [*t*_0_, *β*] and (*d* ^2^/*dt* ^2^)(^RL^ *D*_*t*_0__^*α*^ *e* ^*t*^) ≥ 0 on [*β*, *∞*). Hence ^RL^ *D*_*t*_0__^*α*^ *e* ^*t*^ is convex on [*t*_0_, *β*] and is concave on [*β*, +*∞*).

## 4. Conclusions

In this paper, we first investigate the monotonicity of solutions of nonlinear fractional differential equations with the Caputo's derivative. The results we derive are an improvement of the existing results. Meanwhile, several examples are provided to illustrate the applicability of our results.

The main part of this paper is to study the monotonicity, the concavity, and the convexity of the functions ^RL^ *D*_*t*_0__^*α*^ *f*(*t*) and ^*C*^ *D*_*t*_0__^*α*^ *f*(*t*). Based on the relation between the Riemann-Liouville fractional derivative and the Caputo's derivative, we obtain the criteria on the monotonicity, the concavity, and the convexity of the functions ^RL^ *D*_*t*_0__^*α*^ *f*(*t*) and ^*C*^ *D*_*t*_0__^*α*^ *f*(*t*). In the meantime, five examples are given to illustrate the applications of our criteria.
